# Impact of videogame play on the brain's microstructural properties: cross-sectional and longitudinal analyses

**DOI:** 10.1038/mp.2015.193

**Published:** 2016-01-05

**Authors:** H Takeuchi, Y Taki, H Hashizume, K Asano, M Asano, Y Sassa, S Yokota, Y Kotozaki, R Nouchi, R Kawashima

**Affiliations:** 1 Division of Developmental Cognitive Neuroscience, Institute of Development, Aging and Cancer, Tohoku University, Sendai, Japan; 2 Division of Medical Neuroimaging Analysis, Department of Community Medical Supports, Tohoku Medical Megabank Organization, Tohoku University, Sendai, Japan; 3 Department of Nuclear Medicine and Radiology, Institute of Development, Aging and Cancer, Tohoku University, Sendai, Japan; 4 Research Administration Office, Kyoto University, Kyoto, Japan; 5 Department of Neurology, Medical-Industry Translational Research Center, Fukushima Medical University School of Medicine, Fukushima, Japan; 6 Department of Child and Adolescent Mental Health, National Center of Neurology and Psychiatry, Tokyo, Japan; 7 Division of Clinical Research, Medical-Industry Translational Research Center, Fukushima Medical University School of Medicine, Fukushima, Japan; 8 Human and Social Response Research Division, International Research Institute of Disaster Science, Tohoku University, Sendai, Japan; 9 Department of Functional Brain Imaging, Institute of Development, Aging and Cancer, Tohoku University, Sendai, Japan; 10 Smart Ageing International Research Centre, Institute of Development, Aging and Cancer, Tohoku University, Sendai, Japan

## Abstract

Videogame play (VGP) has been associated with numerous preferred and non-preferred effects. However, the effects of VGP on the development of microstructural properties in children, particularly those associated with negative psychological consequences of VGP, have not been identified to date. The purpose of this study was to investigate this issue through cross-sectional and longitudinal prospective analyses. In the present study of humans, we used the diffusion tensor imaging mean diffusivity (MD) measurement to measure microstructural properties and examined cross-sectional correlations with the amount of VGP in 114 boys and 126 girls. We also assessed correlations between the amount of VGP and longitudinal changes in MD that developed after 3.0±0.3 (s.d.) years in 95 boys and 94 girls. After correcting for confounding factors, we found that the amount of VGP was associated with increased MD in the left middle, inferior and orbital frontal cortex; left pallidum; left putamen; left hippocampus; left caudate; right putamen; right insula; and thalamus in both cross-sectional and longitudinal analyses. Regardless of intelligence quotient type, higher MD in the areas of the left thalamus, left hippocampus, left putamen, left insula and left Heschl gyrus was associated with lower intelligence. We also confirmed an association between the amount of VGP and decreased verbal intelligence in both cross-sectional and longitudinal analyses. In conclusion, increased VGP is directly or indirectly associated with delayed development of the microstructure in extensive brain regions and verbal intelligence.

## Introduction

Videogame play (VGP) is increasingly prevalent among children in the modern era.^1^ VGP has been associated with numerous preferred and non-preferred effects. A causal relationship between VGP and improvements in certain types of visual cognition has been relatively well established.^2^ On the other hand, negative effects of VGP include effects on verbal memory, some types of attention, sleep, learning and knowledge.^2, 3, 4^ Furthermore, in imaging studies, VGP was shown to cause substantial dopamine release in the dopaminergic system^5^ as well as addiction.^6^

Previous cross-sectional studies have revealed that children who play large amounts of videogame and professional online gamers exhibited increased cortical thickness and regional gray matter volume in the dorsolateral prefrontal cortex (PFC), frontal eye field and similar areas.^7, 8, 9^ However, to date the effects of VGP on the development of microstructural properties in children, particularly those associated with negative psychological consequences of VGP, have not been identified. The purpose of this study was to investigate this issue through cross-sectional and longitudinal prospective analyses. Using a longitudinal prospective, observational study design, we can focus on the negative consequences of VGP such as long-term maldevelopment of verbal functions and changes in the dopamine system owing to long VGP. These issues cannot be ethically and practically investigated in controlled short-term interventional studies.

Mean diffusivity (MD) and fractional anisotropy (FA) measures of diffusion tensor imaging^10^ can measure different brain microstructural properties. In particular, a lower MD reflects a greater tissue density, such as the increased presence of cellular structures. The possible mechanisms to affect MD include capillaries, synapses, spines and macromolecular proteins; properties of myelin, membrane and axon; the shape of neurons or glia; or enhanced tissue organization, but MD is not specifically sensitive to any one of them.^10, 11^ Changes in MD have been shown to be uniquely sensitive to neural plasticity.^11, 12^ In particular, MD in the dopaminergic system has been shown to be quite sensitive to pathological, pharmacological and cognitive differences or changes related to dopamine.^12, 13, 14, 15^ On the other hand, FA is known to be relatively more strongly associated with microstructural properties related to brain connectivity and sensitive to increases in axonal membrane thickness, diameter and/or the amount of parallel organization of the axons and can also reflect the process of neural plasticity.^10, 16^ We, therefore, utilized these measures in this study.

Based on the aforementioned previous psychological and neuroimaging studies of VGP, we hypothesized that VGP affects these neural mechanisms in the areas of the PFC and left superior temporal and inferior frontal gyrus, which are involved in verbal processes;^17^ the orbitofrontal and subcortical dopaminergic systems, which are involved in reward and motivational processes;^18^ and the hippocampus, which is involved in memory and sleep.^19^ Given the prevalence of VGP among children, it is important to reveal the consequences of VGP.

## Materials and methods

### Subjects

All subjects were healthy Japanese children. For full descriptions, see Supplementary Methods. As per the Declaration of Helsinki (1991), written informed consent was obtained from each subject and his/her parent. Approval for these experiments was obtained from the Institutional Review Board of Tohoku University. A few years (for details about this interval, see Table 1) after the preexperiment, postexperiment was conducted and part of the subjects from the preexperiment also participated in this postexperiment.

Cross-sectional imaging analyses were performed in 240 subjects (114 boys and 126 girls; mean age, 11.5±3.1 years; range, 5.7–18.4 years), and longitudinal imaging analyses were performed in 189 subjects (95 boys and 94 girls; mean age, 14.5±3.0 years; range, 8.4–21.3 years).

### Assessments of psychological variables

In both the preexperiment and postexperiment, we measured the Full Scale intelligence quotient (FSIQ) using the Japanese version of the Wechsler Adult Intelligence Scale-Third Edition (WAIS-III) for subjects aged ⩾16 years or the Wechsler Intelligence Scale for Children-Third Edition (WISC-III) for subjects aged <16 years.^20^ The tests were administered by trained examiners.^21^ We calculated the FSIQ, verbal IQ (VIQ) and performance IQ (PIQ) for each subject from their WAIS/WISC scores. The Wechsler IQ test is the one of most widely used psychometric measures of cognitive function, and scores of this test reliably predict diverse outcomes in education, career and social relationships.^22^ For the quality check, the correlations of preexperiment test scores with both postexperiment test scores and preexperiment total intracranial volume were calculated (provided in Supplementary Results).

In the preexperiment, the duration of VGP during weekdays was collected using a self-report questionnaire with multiple choice questions. There were the following eight options: 1, none; 2, a little; 3, approximately 30 min; 4, approximately 1 h; 5, approximately 2 h; 6, approximately 3 h; 7, ⩾4 h; and 8, have no way of telling. These choices were transformed into hours of VGP (choice 1=0, choice 2=0.25, choice 3=0.5, choice 4=1, choice 5=2, choice 6=3, choice 7=4) and hours of VGP were used in the statistical analyses described below. Data from subjects who chose option 8 were removed from the analyses involving hours of VGP. This method seems to be a crude way for assessing the amount of VGP. However, it is widely used and has been validated in the field (see Discussion and References of method validity in Supplementary Material).

Further, as additional covariates, we gathered the following information: relationship with parents, number of parents who live together with children, family's annual income, educational qualifications of both parents, and urbanicity of the place (at the municipal level) where the subjects lived. For details about these measures including the detailed methods of assessment, please see our previous study.^23^

For participants in the fourth grade or below, the parents answered questions regarding the amount of VGP and the relationship between children and parents. For participants in the fifth grade or above, children themselves answered these questions. For the rationale for this choice of threshold, see Supplementary Methods.

### Behavioral data analysis

Behavioral data were analyzed using predictive analysis software release version 22.0.0 (PASW Statistics 22; SPSS, Chicago, IL, USA; 2010). For psychological analyses, one-tailed multiple regression analyses were used to investigate the hypothesized negative associations between the amount of VGP and VIQ in the preexperiment (cross-sectional analyses) as well as negative associations between the amount of VGP in the preexperiment and VIQ changes from preexperiment to postexperiment (longitudinal analyses). In the cross-sectional analyses, sex, age (days after birth), family's annual income, average number of years for parents' highest educational qualification, person who answered the question regarding the amount of VGP, urbanicity of the area in which the participant lived, number of parents who lived together with the participant and relationship with parents were added as covariates. Additionally, in longitudinal analyses, the time interval between the preexperiment and postexperiment and the dependent variable of the cross-sectional analysis (VIQ) were added as covariates. Other IQ test scores were investigated in the same manner. One-tailed tests were used for analyses that tested specific hypotheses (negative effects of VGP on VIQ). This was carried out because in these analyses the hypotheses to be tested concerned whether VGP negatively impacts verbal functions. Furthermore, for IQ scores that demonstrated the effects of VGP in cross-sectional analyses, one-tailed tests were used in longitudinal analyses (according to the same directions as those of the effects in cross-sectional analyses).

Multiple comparison corrections were applied to the analytical results that were relevant to the study purpose. In these six analyses, results with a threshold of *P*<0.05 (corrected for false discovery rate (FDR) using the two-stage sharpened method^24^) were considered statistically significant. We considered the results to be significant only when the uncorrected and corrected *P*-values were both <0.05.^25^

### Image acquisition and analysis

Magnetic resonance imaging (MRI) data acquisition was conducted using a 3-T Philips Achieva scanner (Best, The Netherlands). Using a spin-echo echo-planar imaging sequence (TR=10 293 ms, TE=55 ms, *Δ*=26.3 ms, *δ*=12.2 ms, FOV=22.4 cm, 2 × 2 × 2 mm^3^ voxels, 60 slices, SENSE reduction factor=2, number of acquisitions=1), diffusion-weighted data were collected. The diffusion weighting was isotropically distributed along 32 directions (*b*-value=1000 s mm^−2^). Additionally, a single image with no diffusion weighting (*b*-value=0 s mm^−^^2^; *b*0 image) was acquired. The total scan time was 7 min 17 s. FA and MD maps were calculated from the collected images using a commercially available diffusion tensor analysis package on the MR consol. For more details, see Supplementary Methods.

### Preprocessing of imaging data

Preprocessing and analysis of imaging data were performed using SPM8 implemented in Matlab. Basically, we normalized pre- and post-MD and pre- and post-FA images of subjects with previously validated diffeomorphic anatomical registration through exponentiated lie algebra (DARTEL)-based registration process method, then normalized MD images were masked by the custom mask image that is strongly likely to be gray or white matter, and normalized FA images were masked by the custom mask image that is strongly likely to be white matter and smoothed. For details, see Supplementary Methods.

Finally, the signal change in MD (or FA) between the preexperiment and postexperiment images was computed at each voxel within the abovementioned mask for each participant. The resulting maps representing the MD (or FA) change between the pre- and post-MRI experiments ((MD after−MD before) or (FA after−FA before)) were then forwarded to the longitudinal imaging analyses, as described in the following section.

### Whole-brain imaging data analysis

Statistical analyses of the cross-sectional whole-brain imaging data were performed using SPM8. Cross-sectional whole-brain multiple regression analysis was performed to investigate the association between MD or FA and the amount of VGP. The covariates were the same as those used in the psychological cross-sectional analyses, except that in imaging analyses, the total intracranial volume calculated using voxel-based morphometry (for details, see Takeuchi *et al.*^26^) was added as a covariate.

In the longitudinal analyses of MD (or FA), maps representing signal changes in MD (or FA) between the preexperiment and postexperiment images were analyzed. We investigated the association between preexperiment and postexperiment MD (and FA) changes and hours of VGP. The covariates were the same as those used in the psychological longitudinal analyses, except that in imaging analyses, total intracranial volume was added as a covariate and this was made possible by voxel-by-voxel basis using biological parametric mapping tool (BPM) (www.fmri.wfubmc.edu).

The analyses of MD were limited to the gray+white matter mask that was created above. The analyses of FA were limited to the white matter mask that was created above.

A multiple comparison correction of the cross-sectional analyses was performed using threshold-free cluster enhancement (TFCE),^27^ with randomized (5000 permutations) nonparametric permutation testing via the TFCE toolbox (http://dbm.neuro.uni-jena.de/tfce/). We applied the threshold of an family-wise error (FWE)-corrected *P*<0.05. In longitudinal analyses, multiple comparison correction was performed using the FDR approach,^28^ and areas that surpassed the extent threshold^29^ based on this cluster-determining threshold were reported. Different statistical thresholds were taken because (1) permutation tests can generally properly control false positive rates^30^ but (2) BPM does not allow the use of TFCE. We chose the best available statistical method for each analysis.

## Results

### Basic data

The characteristics of the subjects are shown in Table 1. The duration of VGP during weekdays was collected by self-report questionnaire, and the averages and s.ds. are presented in Table 1.

### Cross-sectional behavioral analysis

**Figure 1:**
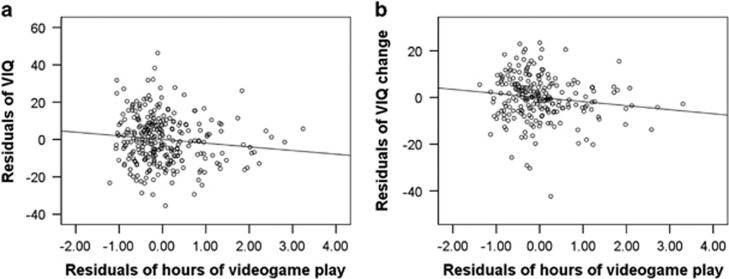
Associations between amount of time (hours) in videogame play (VGP) and verbal intelligence quotient (VIQ) as well as changes across time. (**a**) Partial regression plots with trend lines depicting the correlations between residuals in the multiple regression analyses with VIQ in the preexperiment as a dependent variable and hours of VGP in the preexperiment and other confounding factors as independent variables. (**b**) Partial regression plots with trend lines depicting the correlations between residuals in the multiple regression analyses with longitudinal changes in VIQ as the dependent variable and hours of VGP in the preexperiment and other confounding factors as independent variables.

Multiple regression analyses that used preexperiment data and corrected for confounding variables (see Methods for details) were employed. These analyses revealed that the amount of VGP in the preexperiment was significantly and negatively correlated with VIQ in the preexperiment (Figure 1a, *P*=0.027, uncorrected, *P*=0.038, corrected for FDR, *t*=−1.930, standardized partial regression coefficient (*β*)=−0.120), as expected, and with FSIQ in the preexperiment (*P*=0.032, uncorrected, *P*=0.038, corrected for FDR, *t*=−2.159, *β*=−0.135) but only tended to negatively correlate with PIQ in the preexperiment (*P*=0.061, *P*=0.038, corrected for FDR, *t*=−1.879, *β*=−0.118).

### Longitudinal behavioral analysis

Multiple regression analyses that used longitudinal data and corrected for the confounding variables (see Methods for details) were employed. The results revealed that the hours of VGP in the preexperiment were significantly and negatively correlated with the VIQ change between the preexperiment and postexperiment data (Figure 1b, *P*=0.044, uncorrected, *P*=0.038, corrected for FDR, *t*=−1.710, standardized partial regression coefficient (*β*)=−0.119) but only tended to correlate negatively with FSIQ in the preexperiment with the FSIQ change between the preexperiment and postexperiment data (*P*=0. 064, *P*=0.038, corrected for FDR, *t*=−1.525, *β*=−0.076) and did not correlate with the change in PIQ between the preexperiment and postexperiment data (*P*=0. 595, *P*=0.2975, corrected for FDR, *t*=−0.533, *β*=−0.037).

### Cross-sectional analyses of MD and FA

**Figure 2:**
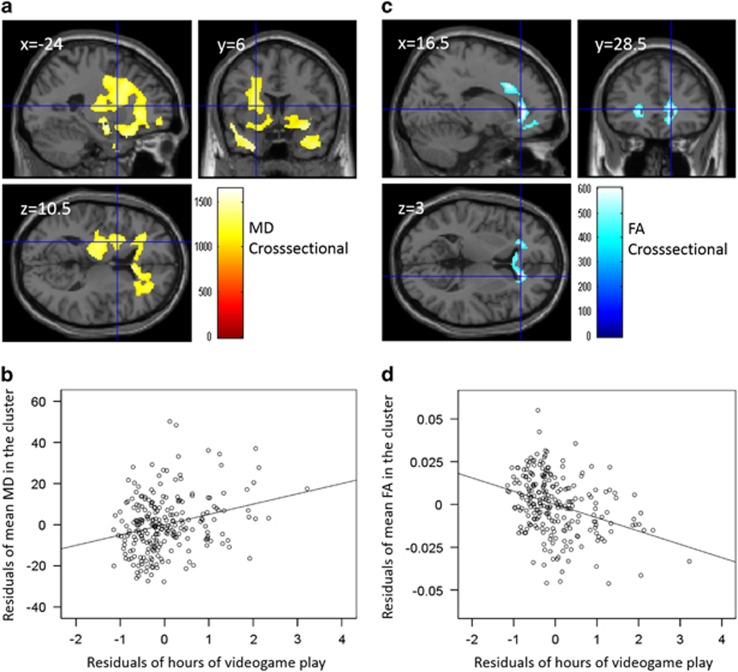
Microstructural property correlates of the amount of time (hours) spent in videogame play (VGP) in cross-sectional analyses (in preexperiments). (**a** and **c**) The results shown were obtained using a threshold of threshold-free cluster enhancement (TFCE) of *P*<0.05, based on 5000 permutations. The results were corrected at the whole-brain level. Regions with significant correlations are overlaid on a ‘single subject' T1 image of SPM8. The color represents the strength of the TFCE value. (**a**) Positive mean diffusivity (MD) correlates of the time spent in VGP. Significant positive correlations with MD were observed in extensive gray and white matter regions of the bilateral prefrontal cortex, anterior cingulate, lateral and medial temporal cortex, basal ganglia and fusiform gyrus. (**b**) Partial regression plots with trend lines depicting correlations between residuals in the multiple regression analyses, with mean MD in the cluster of (**a**) in the preexperiment as a dependent variable and hours of VGP in the preexperiment and other confounding factors as independent variables. (**c**) Negative fractional anisotropy (FA) correlates of time spent in VGP. Significant negative correlations with FA were observed mainly in the areas of the genu and body of the corpus callosum, bilateral anterior corona radiate and right superior corona radiate. (**d**) Partial regression plots with trend lines depicting correlations between residuals in multiple regression analyses, with mean MD in the cluster of (**c**) in the preexperiment as a dependent variable and hours of VGP in the preexperiment and other confounding factors as independent variables.

Multiple regression analyses revealed that the hours of VGP in the preexperiment correlated significantly and positively with MD in the preexperiment in extensive regions of gray and white matter in the bilateral PFC, anterior cingulate, lateral and medial temporal cortex, basal ganglia and fusiform gyrus (see Table 2 and Figures 2a and b for precise anatomical areas). Additionally, there were significant negative correlations between the hours of VGP in the preexperiment and FA, mainly in the areas of the genu and body of the corpus callosum, bilateral anterior corona radiate and right superior corona radiata (see Table 3 and Figures 2c and d for precise anatomical areas).

### Longitudinal analyses of MD and FA

**Figure 3:**
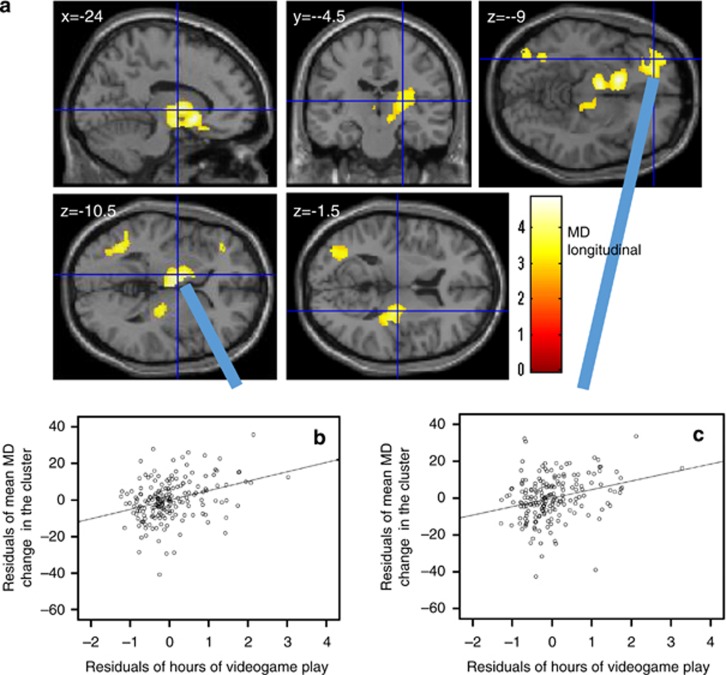
Mean diffusivity (MD) correlates of the amount of time (hours) in videogame play (VGP) in longitudinal analyses. (**a**) Results are shown for a threshold of *P*<0.05 corrected for multiple comparisons in cluster size tests, using a voxel level cluster determining threshold of *P*<0.05 (corrected for false discovery rate). Results were corrected at the whole-brain level. Regions with significant correlations are overlaid on a ‘single subject' T1 image of SPM5. The color represents the strength of the T value. Positive changes in MD correlates of the time spent in VGP. Significant positive correlations with changes in MD were observed in clusters spread throughout gray and white matter areas of the left basal ganglia, left medial temporal, bilateral thalamus, ventral parts of the prefrontal cortex, right insula, left middle and inferior temporal, fusiform and left occipital lobe. (**b** and **c**) Partial regression plots with trend lines depicting correlations between residuals in multiple regression analyses, with mean longitudinal changes in MD of (**b**) the anatomical cluster, including gray and white matter areas of the left basal ganglia, left medial temporal and bilateral thalamus, and of (**c**) a cluster in the ventral parts of the prefrontal cortex as the dependent variables and hours of VGP in the preexperiment and other confounding factors as independent variables.

Multiple regression analyses revealed that the hours of VGP in the preexperiment correlated significantly and positively with changes in MD between the preexperiment and postexperiment in the anatomical cluster that included gray and white matter areas of the left basal ganglia, left medial temporal lobe and bilateral thalamus; a cluster in the ventral parts of the PFC; an anatomical cluster including the gray and white mater areas of the right insula, right putamen and right thalamus; and an anatomical cluster that included gray and white matter areas of the left middle and inferior temporal, fusiform and left occipital lobes (Figures 3a–c, Table 4). There were no significant results associated with FA changes.

### Analyses of MD and psychometric intelligence

**Figure 4:**
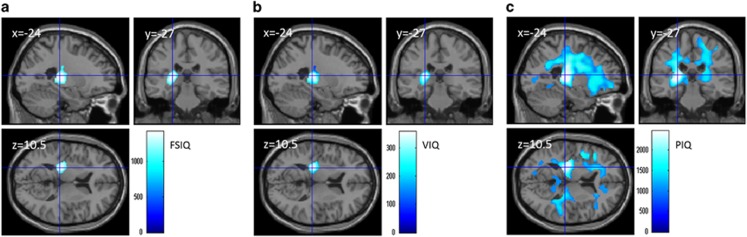
Mean diffusivity (MD) correlates of psychometric intelligence in cross-sectional analyses (in preexperiments). (**a**–**c**) The results shown were obtained using a threshold of threshold-free cluster enhancement (TFCE) of *P*<0.05, based on 5000 permutations. Regions with significant correlations are overlaid on a ‘single subject' T1 image of SPM8. The color represents the strength of the TFCE value. (**a**) Negative MD correlates of Full Scale intelligence quotient (FSIQ). Significant negative correlations with MD were observed in areas mainly around the left thalamus, left hippocampus, left putamen, left insula, left Heschl gyrus and associated white matter bundles, such as the fornix, left superior corona radiate and left internal capsule. The results were corrected at the whole-brain level. (**b**) Negative MD correlates of verbal IQ (VIQ). Significant negative correlations with MD were observed in areas mainly around the left thalamus, left hippocampus, left putamen, left insula, left Heschl gyrus and associated white matter bundles, such as the fornix, left superior corona radiate and left internal capsule. The results were corrected within the areas of significant correlation between MD and FSIQ in Figure 3a. (**c**) Negative MD correlates of performance IQ (PIQ). Significant negative correlations with MD were observed in extensive areas throughout the brain. The results were corrected at the whole-brain level.

Multiple regression analyses that used preexperiment data and corrected for confounding variables (see Supplementary Methods for details) were employed. These analyses revealed that FSIQ correlated significantly and negatively with MD in areas mainly around the left thalamus, left hippocampus, left putamen, left insula, left Heschl gyrus and associated white matter bundles, such as the fornix, left superior corona radiate and left internal capsule (Figure 4a; TFCE value=1423.1, TFCE-corrected *P*-value=0.0166, cluster size=1512 voxels). Further, PIQ significantly and negatively correlated with MD in widespread gray and white matter areas of the widespread areas around the entire brain (Figure 4c; see Supplementary Table S5 for precise anatomical areas). VIQ did not significantly correlate with MD in the whole-brain analysis. However, a substantial trend was observed in areas where the effects of FSIQ were seen. The region of interest analysis revealed that, within this area, VIQ significantly and negatively correlated with MD (Figure 4b; TFCE value=357.31, TFCE-corrected *P*-value=0.002, cluster size=1475 voxels) (for consideration of the statistical validity of this region of interest analysis and the demonstration that the associations between MD and VIQ as well as PIQ in this area are formed by the associations between MD and common components of VIQ and PIQ, see Supplementary Methods and Supplementary Results). These results suggest that PIQ associated with MD in widespread areas and that VIQ associated with a more confined area in the left hemisphere. Additionally, a common effect of PIQ and VIQ led to the effect of FSIQ on MD in this area.

The observed MD correlations with FSIQ and VIQ overlapped with those of VGP in the cross-sectional analyses but not with those in the longitudinal analyses. However, when the threshold for cluster formation was loosened to *P*<0.1 corrected in FDR in the longitudinal analyses of VGP, the formed cluster overlapped the MD correlates of FSIQ and VIQ.

## Discussion

In this study, we have revealed for the first time the effects of VGP on MD and FA in children. Our hypotheses were partly confirmed, and our cross-sectional and longitudinal studies consistently revealed that a greater amount of VGP was associated with increased MD in cortical and subcortical areas and lowered verbal intelligence.

The present MD results and convergent evidence suggest that excessive VGP directly or indirectly disrupts the development of preferable neural systems, which may be related to the delayed development of verbal intelligence. The present results showed that longer VGP is associated with greater MD in extensive regions and lower verbal intelligence, both cross-sectionally and longitudinally. On the other hand, during development, MD generally decreases.^31^ Furthermore, in the present study, a higher PIQ was associated with lower MD in extensive regions in the brain, and higher FSIQ and VIQ were both associated with lower MD in the left thalamus, left hippocampus, left putamen, left insula, left Heschl gyrus and associated white matter bundles. MD in areas including or adjacent to these areas demonstrated the positive effects of VGP both cross-sectionally and longitudinally. These lines of evidence suggest that excessive VGP directly or indirectly disrupts the development of preferable neural systems, which may be related to the delayed development of verbal intelligence.

Previous studies have suggested several physiological mechanisms underlying MD changes. Decreased MD has been suggested to reflect various cellular and cytoarchitectonic changes resulting in higher tissue density, as described in the Introduction section. Further, MD has been shown as uniquely sensitive to neural plasticity, and the abovementioned tissue mechanisms have been shown or suggested to change through processes involving neural plasticity.^11^ As such, a decrease in MD is usually thought to reflect an increase of tissue and functional adaptations. However, MD is not very specific to any particular tissue.^32^ Additionally, MD can reflect blood flow decreases, and in certain cases, functional adaptation is reflected by an increase in MD.^12^ Therefore, whether the decreased MD is an adaptive change should be determined from a comprehensive perspective that includes psychological measures.

All of the identified areas where MD correlated with the amount of VGP in both cross-sectional and longitudinal analyses have been suggested to have unique roles in verbal, memory and executive processes; reward and motivation; and reading and language processes, and through these processes VGP may directly or indirectly lead to previously reported functional deficits. First, the hippocampus is associated with memory and sleep processes.^19^ VGP is known to associate with sleep abnormalities and disturbances in learning, memory and knowledge.^3, 4^ Observed abnormalities in this area that are related to VGP may be associated with deficits in the functions related to VGP. Second, the left middle frontal and inferior frontal gyrus have critical roles in executive functions and the central system and subsystems of the working memory.^33^ On the other hand, these processes are causally disturbed by VGP.^2^ Third, areas in the basal ganglia, orbitofrontal cortex and insula have various roles in reward and motivation processes.^34, 35^ Interestingly, similar to psychostimulants, VGP causes substantial dopamine release in the dopaminergic system^5^ and causes addiction.^6^ Dopamine has been known to exhibit neurotoxic properties, and excessive dopamine damages tissues and cells in the brain.^36^ In addition, a previous study of psychostimulant (methamphetamine) users revealed higher MD in regions of the dopaminergic system.^37^ Further, an intervention study of Parkinson's disease revealed that administration of the dopamine agonist L-dopa led to increased MD in regions of the dopaminergic system.^14^ Therefore, a greater amount of VGP and concomitant increase in dopamine release are associated with later MD changes in the dopaminergic system, akin to the effects of substances that release dopamine. The MD of these areas is associated with traits with negative affect, whereas excessive VGP is associated with emptiness or depressive tendencies when not playing videogames.^38^ Through neural mechanisms in these areas, VGP may be directly or indirectly associated with the previously reported functional deficits. In addition, in the present study, VIQ decreased in response to VGP, and irrespective of IQ type, a lower IQ was associated with higher MD in areas including the dopaminergic system and hippocampus. In addition to learning and memory processes, motivation processes have key roles in IQ test performance among children.^39^ Therefore, the observed effects of VGP on VIQ may be partly mediated by these neural mechanisms. However, these are speculations, as the present study is longitudinal and non-interventional, and we do not have sufficient data to substantiate these speculations and causalities; future studies are needed to confirm these speculations or causalities.

The associations between a greater amount of VGP and lower FA as well as lower PIQ were observed only in cross-sectional analyses. Usually, a lower FA in areas such as the corpus callosum, where multiple neuronal fibers do not cross, is thought to represent non-preferred tract functions that are accompanied by reduced myelination of axons and other physiological mechanisms.^16, 40^ The observed lack of associations in the longitudinal analyses can be attributed to many causes. One is the lower statistical power in longitudinal analyses because of a smaller sample size or increased age, as younger children exhibit greater plasticity.^41^ Also, most prominent plasticity may occur at the initial stage of experience with VGP according to these measures, and neural plasticity may, therefore, not be observed in longitudinal analyses of these measures. The last but most straightforward interpretation is that VGP does not have detectable effects on those measures. The observed cross-sectional association was that children with such neurocognitive characteristics (lower PIQ and lower FA in widespread regions) play videogames in greater amounts. Related to the present findings of FA, previous studies have investigated the FA characteristics of patients with internet addiction.^42, 43^ These studies are relevant to the current results because internet addiction is weakly related to the amount of VGP,^44^ perhaps because of online gaming. Although the findings of these two are inconsistent, one found that patients with internet addiction have lower FA in prefrontal areas, including the anterior parts of the corpus callosum. Further, this study used a questionnaire for children's anxiety-related emotional disorders^45^ and demonstrated that patients with internet addiction exhibit more severe emotional problems and that these problems were associated with FA in the anterior corpus callosum. Although previous studies have shown that the gray matter structural correlates of the amount of VGP were not related to internet addiction,^44^ it is possible that the present FA findings share common pathogenic mechanisms with internet addiction (such as vulnerability and/or acquired signs of addiction/emotional problems). These possibilities should be explored in future studies.

The present studies have advanced our understanding of the direct or indirect effects of VGP in children. As described in previous studies, previous neuroimaging has rather consistently shown a positive correlation between the amount of VGP and amount of gray matter in the DLFPC, and this has generally been considered a positive outcome.^7, 8, 9^ A similar tendency between the amount of VGP and regional gray matter volume in the left dorsolateral PFC *(T*=3.27, 689 mm^3^, *P*<0.0025) was observed in the cross-sectional analysis of this study. In that analysis, VBM analysis was performed using the same covariates used in this study (for details of methods of preprocessing, see Takeuchi *et al.*^26^). However, additional studies have indicated that the increased gray matter related to computer experience in children and young adults exerts negative psychological consequences.^26, 46^ The present studies have investigated the direct or indirect effects of VGP from the perspective of FA and MD and verbal intelligence and has further supported the negative aspects of VGP in younger subjects.

The present study had some limitations. First, this was not an intervention study and, therefore, includes some common limitations of observational epidemiological studies. This study involved longitudinal analyses and was free from some of the limitations (for example, the possibility that associations between verbal intelligence and VGP was caused by a tendency of children with lower intelligence to play videogames). However, the present results still cannot prove that VGP directly caused the observed changes. It is possible that numerous environmental factors that could not be corrected in the analyses caused the observed changes. It is also possible that a reduction in the number of daily activities (for example, studying, reading, conversations with others and exercise) were replaced by time spent in VGP. This is more true in children because children spend their time in a rather uniform manner on weekdays (for example, school). During the remaining time, as certain activities increase, other activities tend to simultaneously decrease. Given this nature, it is not proper to correct for these activities in multiple regression analyses. It should also be remembered that, in children, time spent in VGP reflects a decrease in the time spent on verbal activities (or exercise), and some of the observed effects may have been mediated by such effects. Even if this were the case, we do not think that the purpose of this study was unfulfilled, as the time spent in VGP reflects the nature of time spent in VGP in real life. In other words, unlike experimental settings, in real life even if a certain videogame has beneficial effects on certain functions, substantial time spent playing such a game must replace other favorable activities, such as studying and exercise. For further consideration of this issue and evaluation of the effects of sport, see Supplementary Methods and Results. Further, it is also possible that the amount of VGP was reflective of other impairments (addiction to VGP and low motivation toward academic or social activities) and that such impairments affect neurocognitive functions. Alternatively, when a higher amount of VGP progresses to videogame addiction, this may affect neurocognitive functions. Future studies need to be conducted to consider these causal mechanisms. For further discussions on this issue, see Supplementary Methods. In addition, in this study, we also used a validated and widely used but crude cognitive measure (Wechsler IQ test), and we did not gather data that can specifically evaluate socioemotional measures. The effects of VGP on these specific functions as well as their relation with diffusion tensor imaging measures should be investigated in future studies. Also, studies have shown that certain videogames (for example, violent, spatial and strategic games) have certain specific effects.^47^ Because our study purpose did not address these issues, we did not gather data required to investigate such effects; however, these effects could be studied in the future. One general limitation of this kind of structural study on the effects of environmental factors on neural and cognitive mechanisms is that structural changes do not directly reflect functional changes within the identified areas that are related to cognitive functions. Thus our study cannot directly explain how MD correlates of the amount of VGP in the identified areas are associated with the observed cognitive functional correlates of the amount of VGP and other cognitive functions.

In conclusion, increased VGP is directly or indirectly associated with delayed development of the MD in extensive regions in the brain as well as verbal intelligence. Previously, a wide range of beneficial effects of VGP has been reported,^48^ and videogames may be useful in certain conditions (for example, older adults, certain types of games). However, the present study advanced our understanding of VGP as a daily habit of children and revealed that the conditions in which children play videogames for long periods of time may lead to unfavorable neurocognitive development, at least from a certain perspective.

## Figures and Tables

**Table 1:** Psychological variables of the study participants (mean±s.d., range) in the cross-sectional analyses (114 boys and 126 girls, upper lines) and their change in the longitudinal analyses (95 boys and 94 girls, lower lines when there are two lines)

*Measure*	*Boys*	*Girls*
Age (years) (mean±s.d., range)	11.1±2.7, 5.7 to 16.6 3.0±0.3, 1.7 to 4.0	11.9±3.3, 5.8 to 18.4 3.0±0.3, 1.8 to 4.1
FSIQ (mean±s.d., range)	103.7±12.8, 77 to 134 1.1±8.9, −18 to 24	100.9±11.0, 71 to 128 2.0±9.7, −44 to 26
VIQ (mean±s.d., range)	105.7±13.4, 72 to 152 −0.2±10.3, −27 to 27	102.3±13.0, 67 to 134 1.3±10.3, −41 to 22
PIQ (mean±s.d., range)	100.8±13.0, 62 to 129 2.2±9.9, −23 to 26	98.9±10.7, 73 to 129 2.5±11.1, −50 to 32
Hours of VGP^a^	0.9±0.7, 0 to 3	0.7±0.8, 0 to 4

Abbreviations: FSIQ, full scale intelligence quotient; PIQ, performance intelligence quotient; VGP, videogame play; VIQ, verbal intelligence quotient.

a For the value classification of these measures, see the Methods and Materials section.

**Table 2:** Brain regions that exhibited significant positive effects related to the amount of VGP on MD

*Included gray matter areas*^a^ *(number of significant voxels in the left and right side of each anatomical area)*	*Included large bundles*^b^ *(number of significant voxels in the left and right side of each anatomical area)*	x	y	z	*TFCE value*	*Corrected* P-*value (FWE)*	*Cluster size (voxel)*
Amygdala (L:208, R:378)/caudate (L:133, R:253)/anterior cingulum (L:15, R:480)/inferior frontal operculum (L:133)/inferior frontal orbital area (L:1093, R:607)/inferior frontal triangular (L:467, R:232)/middle frontal medial area (L:107, R:794)/middle frontal orbital area (L:275, R:536)/middle frontal other areas (L:635, R:376)/superior frontal medial area (L:129, R:62)/superior frontal orbital area (L:505, R:793)/superior frontal other areas (R:46)/fusiform gyrus (L:158, R:135)/hippocampus (L:228, R:260)/insula (LL:511:, R:253)/pallidum (L:162, R:48)/parahippocampal gyrus (L:26, R:187)/precentral gyrus (L:97)/putamen (L:717, R:384)/rectus gyrus (L:1, R:123)/rolandic operculum (L:17)/supplemental motor area (L:2)/inferior temporal gyrus (L:679,R:290)/middle temporal gyrus (L:447, R:124)/superior temporal gyrus (L:41, R:2)/temporal pole (L:359, R:430)/thalamus (L:640)	Genu of corpus callosum (1163)/body of corpus callosum (322)/cerebral peduncle (L:131)/anterior limb of internal capsule (L:365, R:86)/posterior limb of internal capsule (L:605)/retrolenticular part of internal capsule (L:19)/anterior corona radiata (L:1440, R:891)/superior corona radiata (L:1041)/sagittal stratum (L:16, R:20)/external capsule (L:304)/cingulate gyrus (R:10)/stria terminalis (L:33, R:34)/superior longitudinal fasciculus (L:351)/superior fronto-occipital fasciculus (L:136)/inferior fronto-occipital fasciculus (L:192, R:281)/uncinate fasciculus (L:111, R:132)	40.5	40.5	−9	1651.33	0.015	27 039

Abbreviations: FWE, family-wise error; MD, mean diffusivity; TFCE, threshold-free cluster enhancement; VGP, videogame play.

a Labelings of the anatomical regions of gray matter were based on the WFU PickAtlas Tool (http://www.fmri.wfubmc.edu/cms/software#PickAtlas/)^49, 50^ and on the PickAtlas automated anatomical labeling atlas option.^51^ Temporal pole areas included all subregions in the areas of this atlas.

b The anatomical labels and significant clusters of major white matter fibers were determined using the ICBM DTI-81 Atlas (http://www.loni.usc.edu/).

**Table 3:** Brain regions that exhibited significant positive effects on the amount of VGP on FA

*Included large bundles (number of significant voxels in the left and right side of each anatomical area)*	x	y	z	*TFCE value*	*Corrected* P*-value (FWE)*	*Cluster size (voxel)*
Genu of corpus callosum (519)/body of corpus callosum (38)/anterior corona radiata (R:563)/sperior corona radiata (R:51)/	16.5	33	6	602.38	0.008	1372
Genu of corpus callosum (58)/anterior limb of internal capsule (L:5)/anterior corona radiata (L:309)/external capsule (L:26)/inferior fronto-occipital fasciculus (L:9)	−19.5	33	3	544.55	0.012	388

Abbreviations: FA, fractional anisotropy; FWE, family-wise error; TFCE, threshold-free cluster enhancement; VGP, videogame play.

**Table 4:** Brain regions that exhibited significant positive correlations between VGP and changes in MD in longitudinal analyses

*No.*	*Included gray matter areas (number of significant voxels in the left and right side of each anatomical area)*	*Included large bundles (number of significant voxels in the left and right side of each anatomical area)*	x	y	z	T *score*	*Corrected* P-*value (FDR)*^a^	*Cluster size (voxel, corrected cluster-level* P-*value)*^a^
1	Amygdala (L:6)/caudate (L:285)/superior frontal orbital area (L:19)/hippocampus (L:30)/pallidum (L:304)/putamen (L:296)/rectus gyrus (L:107)/thalamus (L:457)/	Genu of corpus callosum (4)/cerebral peduncle (L:145)/anterior limb of internal capsule (L:285)/posterior limb of internal capsule (L:257)/inferior fronto-occipital fasciculus (L:31)	−13.5	12	−7.5	4.87	0.016	2630, >0.001
2	Inferior frontal orbital area (L:161)/inferior frontal triangular (L:91)/middle frontal orbital area (L:475)/middle frontal other areas (L:5)/superior frontal orbital area (L:225)	Anterior corona radiata (L:18)	−23.5	45	−12	4.24	0.016	1142, >0.001
3	Caudate (R:2)/Heschl gyrus (R:23)/hippocampus (R:1)/insula (R:263)/lingual gyrus (R:1)/putamen (R:24)/rolandic operculum (R:6)/superior temporal gyrus (R:15)/thalamus (R:320)	Cerebral peduncle (R:141)/posterior limb of internal capsule (R:524)/retrolenticular part of internal capsule (R:310)/superior corona radiata (R:129)/posterior corona radiata (R:23)/external capsule (R:178)/superior longitudinal fasciculus (R:75)	25.5	−21	7.5	4.11	0.016	2082, >0.001
4	Fusiform gyrus (L:173)/inferior occipital lobe (L:331)/middle occipital lobe (L:405)/inferior temporal gyrus (L:21)/middle temporal gyrus (L:197)	Posterior thalamic radiation (L:268)/sagittal stratum (L:3)/superior longitudinal fasciculus (L:2)	−40.5	−58.5	−3	4.10	0.016	1805, >0.001

Abbreviations: FDR, false discovery rate; MD, mean diffusivity; VGP, videogame play.

a Only the clusters that surpassed the extent threshold with the voxel level cluster determining the threshold (*P*<0.05, corrected for the false discovery rate) were noted.
