# Supplementary material for: Impact of videogame play on the brain's microstructural properties: cross-sectional and longitudinal analyses
Source: Mol Psychiatry. 2016 Jan 5;21(12):1781–9. doi: 10.1038/mp.2015.193 (PMC5116480; doi:10.1038/mp.2015.193)
Supplement: Supplementary Information [file mp2015193x1.doc]

**Supplementary online material**

**Supplemental Methods.**

**Subjects.** All subjects were healthy Japanese children and the details related to their initial recruitment (pre-experiment) were described elsewhere [^1^](#_ENREF_1). As described previously, in brief, we successfully collected brain magnetic resonance (MR) images from subjects who did not have any history of malignant tumors or head traumas involving loss of consciousness. Further, based on the self-report, children with a history of epilepsy, impaired color vision, diagnosis of developmental disorders, routine visits to a hospital because of illness, congenital disorders, or routine medications (except daily drugs such as cold or anti-allergy medications) were excluded during the recruitment processes. We did not use specific diagnostic tools, though the second author is a radiologist and thoroughly checked the T1 weighted structural images for unfound neurological diseases. We stipulated that only right-handed children could participate in the study in an advertisement used for subject recruitment and also confirmed that all subjects were right-handed using the self-report questionnaire, the “Edinburgh Handedness Inventory”[^4^](#_ENREF_4). As per the Declaration of Helsinki (1991), written informed consent was obtained from each subject and his/her parent prior to MR scanning after a full explanation of the purpose and procedures of the study was provided. Approval for these experiments was obtained from the Institutional Review Board of Tohoku University. A few years after the pre-experiment (for details about this interval, see Table 1), post-experiment was conducted and part of the subjects from the pre-experiment.

Overall, for the cross-sectional behavioral analyses, effective data were obtained from 283 subjects (137 boys and 146 girls; mean age, 11.1 ± 3.1 years; range, 5.6–18.4 years), and cross-sectional behavioral analyses were performed with these data. Furthermore, for the longitudinal behavioral analyses, effective data were obtained from 223 subjects (115 boys and 108 girls; mean age, 14.2 ± 3.1 years; range, 8.4–21.7 years), and longitudinal behavioral analyses were performed with these data. Because DT images were obtained from only portion of all the subjects, cross-sectional imaging analyses were performed in 240 subjects (114 boys and 126 girls; mean age, 11.5 ± 3.1 years; range, 5.7–18.4 years) and longitudinal imaging analyses were performed in 189 subjects (95 boys and 94 girls; mean age, 14.5 ± 3.0 years; range, 8.4–21.3 years).

**Validity of the method to assess the amount of videogame play**

In this study, we used seemingly a crude method to measure the amount of VGP. Here we demonstrate that this standard method is valid for the purposes of our study.

There are numerous methods to study the effects of videogame play (VGP) in cross-sectional studies, such as to focus on professional game players. However, as far as we are aware, to measure the amount of daily VGP, a simple self-report based on multi-option type questions is most widely used. The general validity of this type of recall-based time estimation method was demonstrated in a previous study [^5^](#_ENREF_5). Further, there are various subtypes of self-reports distinguished, for example, by whether the questions ask the amount of VGP on holidays, or by the number and contents of the question options. However, neuroimaging studies on effects of the amount of VGP have mainly used self-reports based on multi-option type questions . Moreover, psychological studies conducted by leading experts on the effects of VGP have also used these types of questions . Through these studies, the authenticity of the results has been well validated.

Further, a previous two-year longitudinal study used several types of questions related to VGP, including multi-option questions that asked the number of hours of VGP on weekdays, extent of exposure to aggressive games, and frequency of visits to gaming centers [^10^](#_ENREF_10). This study showed that the amount of VGP on weekdays was the most reliable future predictor of becoming pathologically excessive gamers, which lead to socioemotional problems [^10^](#_ENREF_10).

The importance and focus of the effects of simple amount of VGP in this research area is as described. On the other hand, previous studies have also examined the psychological effects of the level of violence in videogames, the types of videogames played (such as action videogames), the amount of multi-player video gaming, and the frequency of online gaming. Considering all these factors is beyond our current capability as well as statistical power, but future studies are warranted to elucidate the effects of these factors on brain microstructure. Our study should be considered a foundation for such investigations. Previous cross-sectional neuroimaging studies on the effects of VGP have usually not been able to investigate these issues . However, one study investigated effects of the amount of general VGP and then subsequently conducted a preliminary analysis (i.e., at an uncorrected statistical level) of the types of videogames most relevant to the effects observed [^11^](#_ENREF_11).

Finally, studies in this area have also investigated the effects of internet addiction on brain structure [^12-14^](#_ENREF_12). We have not gathered relevant information on internet addiction partly because when we planned the current project (several years ago), the problem of online gaming was not as acute among young children in our country as far as we were aware. The effects of videogame addiction and internet gaming addiction on neural structure and function are both important topics. In the neuroimaging field, to date, studies that had investigated effects of media addiction, generally had not assessed the effects of the simple amount of exposure to the same media simultaneously [^12-15^](#_ENREF_12) and vice versa . The strength of the association between videogame addiction and the amount of VGP is so high (close to r = 0.7) [^16^](#_ENREF_16) that it does not allow for the separation of these effects by standard analyses such as multiple regression due to multicollinearity. The strengths of the negative associations between the amount of VGP and academic achievement and between the severity of videogame addiction and academic achievement are almost identical [^16^](#_ENREF_16). The causal model suggests a causal flow in which a higher VGP leads to addictive VGP, which in turn leads to socioemotional problems [^10^](#_ENREF_10). In our project, the primary focus was the effects of daily habits on neurocognitive development and not the neural pathology of addiction, and our purpose was fulfilled in that sense with the current methodological tools. Nonetheless, future studies with a larger sample size are needed to reveal the neural correlates of causal flow between VGP, videogame addiction, and socioemotional problems.

**Rationale for the choice of age threshold determining whether the subject or parent answered the questionnaire**

In this study, for participants in the fourth grade or below, the parents answered questions regarding the amount of VGP and the relationship between children and parents. For participants in the fifth grade or above, children themselves answered these questions. As described previously [^17^](#_ENREF_17), this threshold was based on customs in the field and previous recommendations . For details regarding this rationale, please see our previous work [^17^](#_ENREF_17). All other questions were answered by parents regardless of a child’s grade.

**Details of diffusion image acquisition**

There are acquisitions for phase correction and for signal stabilization and these are not used as reconstructed images. MD and FA maps were calculated from the collected images using a commercially available diffusion tensor analysis package on the MR consol. This practice has been used in many of our previous studies [^20-24^](#_ENREF_20). Furthermore, the results of analyses using these image-generated results were congruent with those of previous studies in which other methods were used , suggesting the validity of this method. These procedures involved correction for motion and distortion caused by eddy currents. Calculations were performed according to a previously proposed method [^27^](#_ENREF_27).

**Preprocessing of imaging data until smoothing**

Preprocessing and analysis of functional activation data were performed using SPM8 implemented in Matlab. First, the skull in the b = 0 image of each participant was stripped as described previously [^23^](#_ENREF_23); using the resulting image, diffusion images were linearly aligned to the skull-stripped b = 0 image template created previously [^23^](#_ENREF_23) to assist with the following procedures.

Subsequently, using a previously validated two-step new segmentation algorithm of diffusion images and the previously validated diffeomorphic anatomical registration through exponentiated lie algebra (DARTEL)-based registration process [^21^](#_ENREF_21), all images, including gray matter segment [regional gray matter density (rGMD) map], white matter segment [regional white matter density (rWMD) map], cerebrospinal fluid (CSF) segments [regional CSF density (rCSFD) map] of diffusion images, were normalized. The voxel size of these normalized images was 1.5 × 1.5 × 1.5 mm^3^. In these processes, the template for the DARTEL process was created from the pre-experiment images of all subjects whose diffusion imaging data were obtained in the pre-experiment.

Next, we created average images of normalized rGMD and rWMD images of all subjects whose diffusion imaging data were obtained in the pre-experiment. Subsequently, for the analyses of MD images from the normalized images of the (a) MD, (b) rGMD, and (c) rCSFD maps, we created images where areas that were not strongly likely to be gray or white matter in our averaged normalized rGMD and rWMD images (defined by “gray matter tissue probability + white matter tissue probability < 0.99”) were removed (to exclude the strong effects of CSF on MD throughout analyses). These images were then smoothed (8 mm full-width half-maximum) and carried through to the second-level analyses of MD.

Next, we created average from the average image of normalized WM segmentation images of all subjects whose diffusion imaging data were obtained in the pre-experiment. And from the created mask image consisting of voxels with a WM signal intensity > 0.99. We then applied this mask image to the normalized FA image; therefore, we retained only areas that are highly likely to be white matter from the normalized FA images. These images were smoothed (6 mm full-width half-maximum) and carried through to the second-level analyses of FA.

**Interaction effects between age (who answered the question on amount of VGP) and amount of VGP and their impact on the significant correlates of amount of VGP**

To investigate whether the psychological and microstructural correlates with the amount of VGP were associated with a particular age group, we performed ANCOVAs in PASW Statistics 22. The dependent variables in these analyses were those that exhibited significance or a tendency to associate with the amount of VGP in cross-sectional or longitudinal analyses in the present study (namely, one of the following: VIQ in the cross-sectional and longitudinal analyses, FSIQ in the cross-sectional analysis, and mean MD or FA values for significant clusters identified in the cross-sectional and/or longitudinal analyses). In these analyses, the subjects were divided into 2 groups on the basis of age groups (≤4^th^ grade or ≥5^th^ grade, this corresponds to who answered the question on amount of VGP, gurdians or children themselves). We added this group factor as well as the interaction between this group factor and the amount of VGP as covariates in the models for each ANCOVA, in addition to all other covariates (except the covariate of the person who answered the question) used in each of the abovementioned cross-sectional and longitudinal analyses of correlates with the amount of VGP. These analyses are irrelevant to the purpose of this study, and the study was not designed to investigate these effects (such as by recruiting older and younger subjects). Thus, the results were treated in an exploratory manner and corrections of multiple comparisons were not applied.

**About assessing “other activities”.**

As in most previous studies, we did not consider the effects of “other activities” that VGP may have replaced. Here we assess the effects of sports activities, which may have different effects on neural development than other mental activities. However, in doing so, we must explain the validity of our main analyses from several perspectives. First, to our knowledge, such analyses to assess (all or most) other activities that VGP may have replaced were not conducted in any previous studies in this field. Regardless of the neural correlates of VGP , those observed in professional videogamers , or those of internet addiction [^12-14^](#_ENREF_12), if one is so engaged in VGP and media, he or she must tend to spend less time engaged in physical activities or other mental activities. To the best of our knowledge, in psychological studies of VGP, including those of leading experts as well, the effects of physical activities or other mental activities have not been assessed. Second, we must explain why such an assessment is technically and conceptually difficult in this type of observational study of children (for us or anyone in the field). For example, children are exposed to verbal stimuli in countless situations or situations that can potentially improve verbal function. Evaluating all these activities is quite difficult or at least no one has ever attempted to do so to our knowledge. Further, children spend a substantial part of their time at school, and the remaining time is limited. If we list all other activities (other than VGP), the accumulated time will almost equal awake time – school time – the hours of VGP. In other words, the effects of such accumulated time should have effects that are nearly the exact opposite of VGP. Considering this point, we believe conceptually in these kinds of epidemiological studies of the effects of children’s major activities (especially effects that are supposed to include “unuse”-dependent plasticity or loss of functions that are not used), evaluating effects of all (or most) activities that are not the target of the study is not necessary for the purpose of the stud. In addition, in our opinion, the negative effects of VGP on verbal functions are supposed to partly include the conceptual nature that is common with the negative effect of VGP on obesity, in that the effects of videogames are all relative to effects of other activities (e.g., videogame is not good for obesity compared with other major activities such as playing outside). Considering all these factors, we believe that VGP impedes verbal development and related academic achievement in this and previous studies at least partly by reducing verbal activities. Thus, effects that are mediated by such pathways are not necessarily incongruent with our hypothesis nor the purpose of this study.

**The impact of sports activities on the present findings of VGP**

Finally, using available data on children’s participation in sports, we evaluated if sports altered the neural and psychological effects of VGP. Here we used results from an exploratory questionnaire that was available in this project to assess sports experience. In this questionnaire, each item asks how much time (hours in a day and number of times in a week) and for how long subjects engaged in specified activities. The specified sports included soccer, swimming, baseball, ballet, tennis, volleyball, basketball, budo (Japanese martial arts, such as Karate, Judo, Kendo and so on), skiing, and skating. In addition, “other activities” could be specified by the subjects. We calculated the total sum of weekly hours for each sport in the pre-experiment. This measure of sports engagement was only exploratory as it did not assess the intensity of the sport, estimate the consumption of calories, or assess the amount of aerobic exercise. There is a certain limitation.

Next, we assessed whether including the amount of sports participation as a covariate in the multiple regression analyses affected the significant correlations found with the amount of VGP. Here we assessed how much the beta values of the significant correlations changed after including sports participation as a covariate (in the case of imaging analyses, mean imaging values within the significant clusters were used).

**Whole brain analyses for the associations of MD with FSIQ, VIQ, and PIQ**

Next, to reveal the nature of correlations between MD and the amount of VGP in terms of the association with psychometric intelligence (see Results), we investigated the association between FSIQ, VIQ, PIQ, and MD using three voxel-by-voxel multiple regression analyses of MD during the pre-experiment. In these three separate multiple regression analyses, sex, age (days after birth), and one of VIQ, PIQ, or FSIQ were used as independent variables. Here, given the multi-colinearity due to the high correlations among VIQ, PIQ, and FSIQ, three separate multiple regression analyses were employed. Next we performed an additional analysis to address the possibility that significant MD correlates of VIQ in the small volume correction within the area of significant MD correlates of FSIQ is a spurious result of “double dipping”. It has been suggested that “double dipping” was deemed inappropriate in whole brain analyses[^31^](#_ENREF_31). Here this term refers to the situation in which whole brain voxel-based correlation analyses using the the first certain variable (A) was performed first and then significant areas were extracted. Then, the imaging values (B) of this area were shown to correlate with a third behavioral variable (C) that correlates with the first variable (A). In whole brain voxel-based analyses, among many voxels, voxels that showed substantially overestimated effect size because of pure chance can become significant after stringent correction for multiple comparisons (even when the true effect exists in the area around such voxels). To address this problem, we conducted an additional whole brain multiple regression analysis, in which MD is the dependent variable and age, sex, FSIQ, and residual from the simple linear regression of PIQ on FSIQ are independent variables (the variance of PIQ that is not explained by FSIQ). Note that because FSIQ is calculated from the sum of the raw scores of PIQ and VIQ, the residual from the simple linear regression of PIQ on FSIQ and of VIQ on FSIQ should be conceptually opposite, and they are in fact strongly negatively correlated (r = −0.99, simple regression). Thus, in this analysis, among these two residuals, inclusion of only one of them will serve the purpose of analysis. The residual from the simple linear regression of PIQ on FSIQ significantly correlated with PIQ (r = 0.566, P < 0.001, simple linear regression) but not with FSIQ (r was almost 0).

**Supplemental Results**

*Basic data regarding scores of the Wechsler IQ test*

In the study sample, the simple correlation coefficient between pre-experiment FSIQ and pre-experiment VIQ was 0.881 (*P* < 0.001), the simple correlation coefficient between pre-experiment FSIQ and pre-experiment PIQ was 0.828 (*P* < 0.001), and the simple correlation coefficient between pre-experiment VIQ and pre-experiment PIQ was 0.469 (*P* < 0.001). The simple correlation coefficients between the test score of the pre-experiment and that of the post-experiment were 0.695 for VIQ, 0.677 for PIQ, and 0.728 for FSIQ (all P < 0.001). The simple correlation coefficients between the pretest and total intracranial volume (TIV) of the pre-experiment calculated using voxel-based morphometry [(for details, see ref^. 2^](#_ENREF_2)) in this sample were 0.189 for VIQ (P = 0.003), 0.071 for PIQ (P = 0.271), and 0.158 for FSIQ (P = 0.014). Though the simple correlation coefficients between psychometric intelligence and TIV were somewhat smaller than those between brain size and psychometric intelligence previously reported by a meta-analysis (r = 0.33)[^30^](#_ENREF_30), note the possibility of publication bias as well as the fact that the present study sample was composed of children, and apparently, TIV has a larger variability in this cohort than in adult cohorts.

*Interaction effects between age (who answered the question on amount of VGP) and amount of VGP and their impact on the significant correlates of amount of VGP*

We also investigated the interaction effects between age (≤4^th^ grade or ≥5^th^ grade) and the amount of VGP to determine their impacts on the variables that exhibited significance or a tendency toward association with the amount of VGP in both the cross-sectional and longitudinal analyses identified in the present study. In the cross-sectional and longitudinal analyses of psychological and longitudinal imaging correlates of the amount of VGP, no significant interaction effects were observed between the age groups and amount of VGP with respect to the psychological and imaging correlates of amount of VGP (*P* > 0.10, uncorrected).

*Impact of sports activities on effects of VGP*

Results of the analyses including the level of sports participation as a covariate revealed little effect on the strength of the correlations between the amount of VGP and psychological and imaging variables. In some cases, the beta values decreased slightly, and in other cases, the beta values increased, but when the beta values decreased, they did not decrease more than 0.05. These results suggest that the level of sports participation was not a major factor contributing to the present associations with the amount of VGP.

*Results of the whole brain multiple regression analysis, in which MD is the dependent variable and age, sex, FSIQ, and the residual from the simple linear regression of PIQ on FSIQ are independent variables (the variance of PIQ that is not explained by FSIQ)*

The results showed that FSIQ was significantly and negatively correlated with MD in the areas of significant correlation between FSIQ and MD from the analysis presented in the main text (the one that included only age, sex, and FSIQ as covariates and did not include the residual from the simple linear regression of PIQ) (Supplemental Fig. 1a). Further, the residual from the simple regression of PIQ on FSIQ was significantly and negatively correlated with MD across widespread areas of the whole brain (Supplemental Fig. 1b). These correlations are similar to those between MD and PIQ in the analysis presented in the main text except in the areas of significant correlation between FSIQ and MD from the analysis of the main text.

These results suggest that the significant correlation between FSIQ and MD in the analysis presented the main text (the one that did not include the residual from the simple linear regression of PIQ) came from the common components of VIQ and PIQ (FSIQ). Further, the results suggest that deviation from them in PIQ (or VIQ) [the unique variance of PIQ (or VIQ) that is not explained by the common component of VIQ and PIQ (FSIQ)] was associated with MD in the widespread areas.

**References**

1. Taki Y, Hashizume H, Sassa Y, Takeuchi H, Asano M, Asano K *et al.* Breakfast staple types affect brain gray matter volume and cognitive function in healthy children. *PLoS ONE* 2010; **5**(12)**:** e15213.

2. Takeuchi H, Taki Y, Hashizume H, Asano K, Asano M, Sassa Y *et al.* The Impact of Television Viewing on Brain Structures: Cross-Sectional and Longitudinal Analyses. *Cereb Cortex* 2015; **25**(5)**:** 1188-1197.

3. Taki Y, Hashizume H, Thyreau B, Sassa Y, Takeuchi H, Wu K *et al.* Linear and curvilinear correlations of brain gray matter volume and density with age using voxel‐based morphometry with the Akaike information criterion in 291 healthy children. *Human brain mapping* 2013; **34**(8)**:** 1857-1871.

4. Oldfield RC. The assessment and analysis of handedness: the Edinburgh inventory. *Neuropsychologia* 1971; **9**(1)**:** 97-113.

5. Robinson JP. The validity and reliability of diaries versus alternative time use measures. In: Juster FT, Stafford FP (eds). *Time, goods, and well-being*. Institute

for Social Research, The University of Michigan: Ann Arbor, 1985, pp 63-92.

6. Kühn S, Romanowski A, Schilling C, Lorenz R, Mörsen C, Seiferth N *et al.* The neural basis of video gaming. *Translational Psychiatry* 2011; **1**(11)**:** e53.

7. Kühn S, Lorenz R, Banaschewski T, Barker GJ, Büchel C, Conrod PJ *et al.* Positive association of video game playing with left frontal cortical thickness in adolescents. *PLoS ONE* 2014; **9**(3)**:** e91506.

8. Przybylski AK. Electronic gaming and psychosocial adjustment. *Pediatrics* 2014; **134**(3)**:** e716-e722.

9. Desai RA, Krishnan-Sarin S, Cavallo D, Potenza MN. Video-gaming among high school students: health correlates, gender differences, and problematic gaming. *Pediatrics* 2010; **126**(6)**:** e1414-e1424.

10. Gentile DA, Choo H, Liau A, Sim T, Li D, Fung D *et al.* Pathological video game use among youths: a two-year longitudinal study. *Pediatrics* 2011; **127**(2)**:** e319-e329.

11. Kühn S, Gallinat J. Amount of lifetime video gaming is positively associated with entorhinal, hippocampal and occipital volume. *Mol Psychiatry* 2014; **19**(7)**:** 842-847.

12. Lin F, Zhou Y, Du Y, Qin L, Zhao Z, Xu J *et al.* Abnormal white matter integrity in adolescents with internet addiction disorder: a tract-based spatial statistics study. *PLoS ONE* 2012; **7**(1)**:** e30253.

13. Yuan K, Qin W, Wang G, Zeng F, Zhao L, Yang X *et al.* Microstructure abnormalities in adolescents with internet addiction disorder. *PLoS ONE* 2011; **6**(6)**:** e20708.

14. Zhou Y, Lin F-c, Du Y-s, Zhao Z-m, Xu J-R, Lei H. Gray matter abnormalities in Internet addiction: a voxel-based morphometry study. *Eur J Radiol* 2011; **79**(1)**:** 92-95.

15. Li W, Li Y, Yang W, Wei D, Li W, Hitchman G *et al.* Brain structures and functional connectivity associated with individual differences in internet tendency in healthy young adults. *Neuropsychologia* 2015; **70:** 134-144.

16. Brunborg G, Mentzoni R, Frøyland L. Is video gaming, or video game addiction, associated with depression, academic achievement, heavy episodic drinking, or conduct problems? *Journal of behavioral addictions* 2014; **3**(1)**:** 27-32.

17. Takeuchi H, Taki Y, Hashizume H, Asano K, Asano M, Sassa Y *et al.* The impact of parent–child interaction on brain structures: Cross-sectional and longitudinal analyses. *The Journal of Neuroscience* 2015; **35**(5)**:** 2233-2245.

18. Kohl HWI, Fulton JE, Caspersen CJ. Assessment of physical activity among children and adolescents: a review and synthesis. *Prev Med* 2000; **31**(2)**:** S54-S76.

19. Kambara M, Miyashita K, Ohnogi H, Nakazawa J. *Psychology manual, methods of questionnaires (Japanese)*. Kitaohji shobo: Kyoto, 1998.

20. Takeuchi H, Taki Y, Sassa Y, Hashizume H, Sekiguchi A, Nagase T *et al.* White matter structures associated with emotional intelligence: Evidence from diffusion tensor imaging. *Hum Brain Mapp* 2013; **34**(5)**:** 1025-1034.

21. Takeuchi H, Taki Y, Thyreau B, Sassa Y, Hashizume H, Sekiguchi A *et al.* White matter structures associated with empathizing and systemizing in young adults. *Neuroimage* 2013; **77**(15)**:** 222-236.

22. Takeuchi H, Sekiguchi A, Taki Y, Yokoyama S, Yomogida Y, Komuro N *et al.* Training of Working Memory Impacts Structural Connectivity. *J Neurosci* 2010; **30**(9)**:** 3297-3303.

23. Takeuchi H, Taki Y, Sassa Y, Hashizume H, Sekiguchi A, Fukushima A *et al.* White matter structures associated with creativity: Evidence from diffusion tensor imaging. *Neuroimage* 2010; **51**(1)**:** 11-18.

24. Takeuchi H, Taki Y, Sassa Y, Hashizume H, Sekiguchi A, Fukushima A *et al.* Verbal working memory performance correlates with regional white matter structures in the fronto-parietal regions. *Neuropsychologia* 2011; **49**(12)**:** 3466-3473

25. Taki Y, Thyreau B, Hashizume H, Sassa Y, Takeuchi H, Wu K *et al.* Linear and curvilinear correlations of brain white matter volume, fractional anisotropy, and mean diffusivity with age using voxel-based and region of interest analyses in 246 healthy children. *Hum Brain Mapp* 2013; **34**(8)**:** 1842-1856.

26. Barnea-Goraly N, Menon V, Eckert M, Tamm L, Bammer R, Karchemskiy A *et al.* White matter development during childhood and adolescence: a cross-sectional diffusion tensor imaging study. *Cereb Cortex* 2005; **15**(12)**:** 1848-1854.

27. Le Bihan D, Mangin JF, Poupon C, Clark CA, Pappata S, Molko N *et al.* Diffusion tensor imaging: concepts and applications. *Journal of Magnetic Resonance Imaging* 2001; **13**(4)**:** 534-546.

28. Han DH, Lyoo IK, Renshaw PF. Differential regional gray matter volumes in patients with on-line game addiction and professional gamers. *J Psychiatr Res* 2012; **46**(4)**:** 507-515.

29. Hyun GJ, Shin YW, Kim B-N, Cheong JH, Jin SN, Han DH. Increased Cortical Thickness in Professional On-Line Gamers. *Psychiatry investigation* 2013; **10**(4)**:** 388-392.

30. McDaniel MA. Big-brained people are smarter: A meta-analysis of the relationship between in vivo brain volume and intelligence. *Intelligence* 2005; **33**(4)**:** 337-346.

31. Vul E, Harris C, Winkielman P, Pashler H. Reply to comments on “puzzlingly high correlations in fMRI studies of emotion, personality, and social cognition.” *Perspect Psycholo Sci* 2009; **4**(3)**:** 319-324.

32. Takeuchi H, Sugiura M, Sassa Y, Sekiguchi A, Yomogida Y, Taki Y *et al.* Neural correlates of the difference between working memory speed and simple sensorimotor speed: an fMRI study. *PLoS ONE* 2012; **7**(1)**:** e30579.

**Supplemental Table 1**

Brain regions that exhibited significant negative correlations between PIQ and MD

| No | Included gray matter areas (number of significant voxels in left and right side of each anatomical area) | Included large bundles (number of significant voxels in left and right side of each anatomical area) | x | y | z | TFCE value | Corrected p value (FWE) | Cluster size (voxel) |
| --- | --- | --- | --- | --- | --- | --- | --- | --- |
| 1 | Angular gyrus (L:58)/Calcarine Cortex (L:145)/Caudate (L:229)/Anterior cingulum (L:221)/Middle cingulum (L:782)/Cuneus (L:42)/Inferior frontal operculum (L:597)/Inferior frontal orbital area (L:888)/Inferior frontal triangular (L:788)/Middle frontal medial area (L:114)/Middle frontal orbital area (L:459)/Middle frontal other areas (L:469)/Superior frontal medial area (L:613)/Superior frontal orbital area (L:819)/Fusiform gyrus (L:455)/Heschl gyrus (L:46)/Hippocampus (L:214)/Insula (L:1387)/Lingual gyrus (L:79)/Inferior occipital lobe (L:211)/Middle occipital lobe (L:209)/Superior occipital lobe (L:41)/Pallidum (L:5)/Paracentral lobule (L:3)/Inferior parietal lobule (L:46)/Superior parietal lobule (L:1)/Postcentral gyrus (L:726)/Precentral gyrus (L:1475)/Precuneus (L:14)/Putamen (L:717)/Rectus gyrus (L:342)/Rolandic operculum (L:557)/Supplemental motor area (L:690)/Supramarginal gyrus (L:29)/Inferior temporal gyrus (L:116)/Middle temporal gyrus (L:337)/Superior temporal gyrus (L:9)/Thalamus (L:1098)/ | Genu of corpus callosum (136)/Body of corpus callosum (592)/Splenium of corpus callosum (114)/Cerebral peduncle (L:73)/Anterior limb of internal capsule (L:377)/Posterior limb of internal capsule (L:761)/Retrolenticular part of internal capsule (L:782)/Anterior corona radiata (L:2151)/Superior corona radiata (L:2388)/Posterior corona radiata (L:1169)/Posterior thalamic radiation (L:734)/Sagittal stratum (L:139)/External capsule (L:1015)/Cingulum (L:343)/Stria terminalis (L:269)/Superior longitudinal fasciculus (L:1768)/Superior fronto-occipital fasciculus (L:167)/Inferior fronto-occipital fasciculus (L:269)/Tapatum (L:78)/ | -25.5 | -24 | 3 | 2465.14 | 0.003 | 35637 |
| 2 | Angular gyrus (R:6)/Calcarine Cortex (L:78, R:28)/Caudate (R:291)/Anterior cingulum (R:801)/Middle cingulum (R:608)/Posterior cingulum (L:180, R:203)/Cuneus (R:17)/Inferior frontal operculum (R:80)/Inferior frontal orbital area (R:32)/Inferior frontal triangular (R:314)/Middle frontal medial area (R:172)/Middle frontal orbital area (R:215)/Superior frontal medial area (R:207)/Superior frontal orbital area (R:220)/Superior frontal other areas (R:370)/Fusiform gyrus (R:762)/Heschl gyrus (R:12)/Hippocampus (R:69)/Insula (R:221)/Lingual gyrus (L:43, R:280)/Inferior occipital lobe (R:29)/Superior occipital lobe (R:10)/Paracentral lobule (R:233)/Parahippocampal gyrus (R:14)/Inferior parietal lobule (R:8)/Superior parietal lobule (R:24)/Postcentral gyrus (R:604)/Precentral gyrus (R:386)/Precuneus (L:96, R:500)/Putamen (R:402)/Rectus gyrus (R:170)/Rolandic operculum (R:18)/Supplemental motor area (R:391)/Supramarginal gyrus (R:23)/Inferior temporal gyrus (R:217)/Middle temporal gyrus (R:12)/Superior temporal gyrus (R:345)/Thalamus (R:498)/ | Genu of corpus callosum (263)/Body of corpus callosum (513)/Splenium of corpus callosum (1019)/Cerebral peduncle (R:3)/Anterior limb of internal capsule (R:28)/Posterior limb of internal capsule (R:311)/Retrolenticular part of internal capsule (R:615)/Anterior corona radiata (R:1256)/Superior corona radiata (R:1855)/Posterior corona radiata (R:883)/Posterior thalamic radiation (R:576)/Sagittal stratum (R:260)/External capsule (R:298)/Cingulum (L:52, R:180)/Heschl gyrus (L:8, R:8)/Stria terminalis (R:62)/Superior longitudinal fasciculus (R:1070)/Superior fronto-occipital fasciculus (R:37)/Inferior fronto-occipital fasciculus (R:175)/Tapatum (R:5)/ | 37.5 | -49.5 | -10.5 | 1675.25 | 0.010 | 23114 |
| 3 | Middle temporal gyrus (R:67)/Temporal pole (R:214) | Uncinate fasciculus (R:3) | 46.5 | 9 | -27 | 1029.05 | 0.042 | 476 |
| 4 | Middle temporal gyrus (R:7)/ | None | 63 | -33 | -9 | 953.79 | 0.050 | 7 |

**Supplemental Figure legends**

**Supplemental Fig. 1.** Effects of FSIQ and variances of PIQ that is not explained by FSIQ on MD. (a, b) The results shown were obtained using a threshold of threshold-free cluster enhancement (TFCE) of *P* < 0.05, based on 5000 permutations. Regions with significant correlations are overlaid on a “single subject” T1 image of SPM8. The color represents the strength of the TFCE value. (a) Negative correlations between FSIQ and MD in the multiple regression analysis, in which age, sex, and FSIQ and residual from the simple linear regression of PIQ on FSIQ are independent variables. Significant negative correlations with MD were observed in areas of significant correlations of FSIQ, VIQ, and PIQ in Fig.3 of the main text. (b) Negative correlations were observed between “residual from the simple linear regression of PIQ” and MD in the multiple regression analysis, in which age, sex, and FSIQ and residual from the simple linear regression of PIQ on FSIQ are independent variables. Significant negative correlations with MD were observed in areas of significant correlations of PIQ (see Fig. 3) except in the area of Supplemental Fig. 1 (a).

**Supplemental Fig. 1**

**
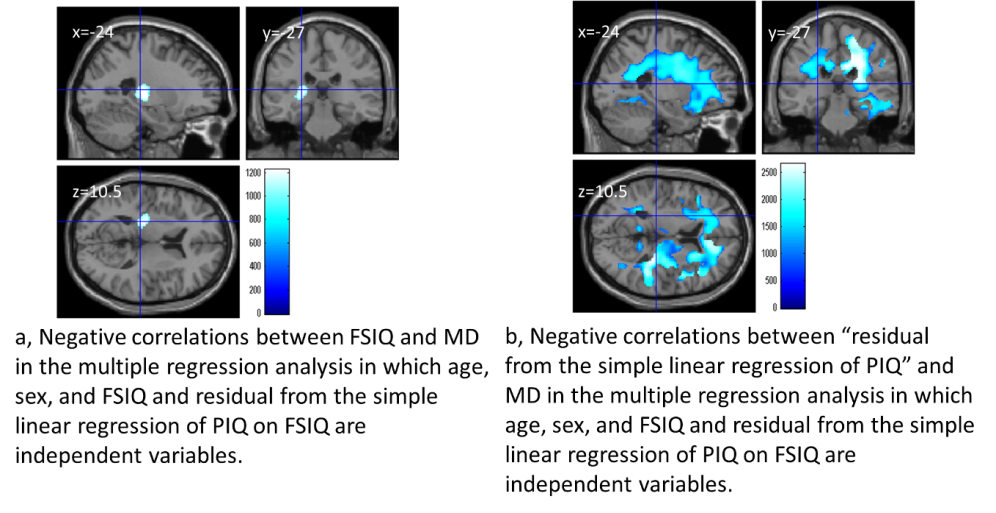
**
