# Supplementary figures and images for: Impact of videogame play on the brain's microstructural properties: cross-sectional and longitudinal analyses
Source: Mol Psychiatry. 2016 Jan 5;21(12):1781–9. doi: 10.1038/mp.2015.193 (PMC5116480; doi:10.1038/mp.2015.193)

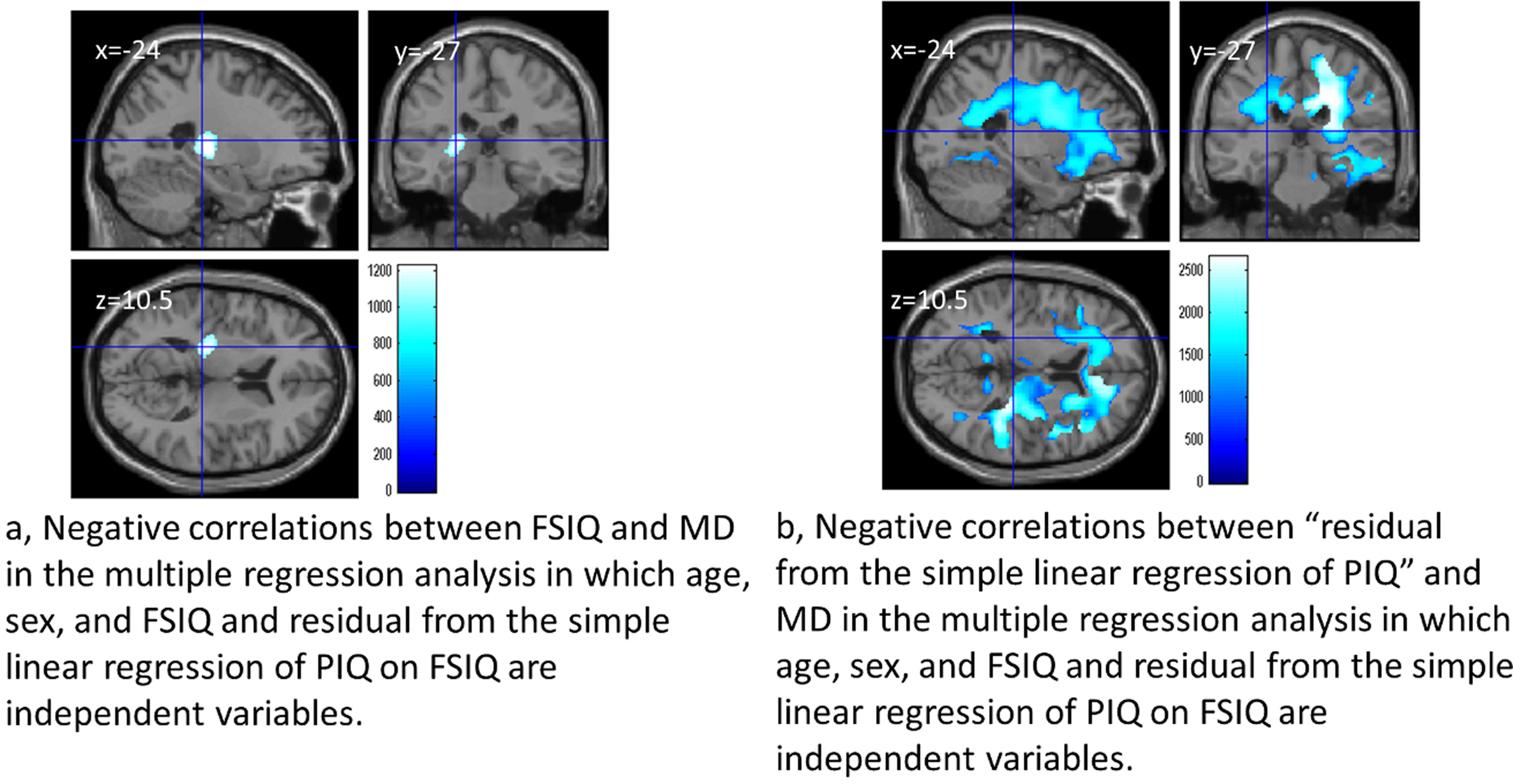

Supplement: Supplementary Figure 1 [file mp2015193x2.tif]
